# Report of Deaths in Buffalo for the Month of June, 1855

**Published:** 1855-08

**Authors:** J. Root

**Affiliations:** Health Physician


### ART. VI.—*Report of Deaths in Buffalo for the month*

| DISEASES. | Males. | Females. | Total. | AGE. Under 1 year. Male. | Under 1 year. Female. | 1 to 2 years. Male. | 1 to 2 years. Female. |
|---|---|---|---|---|---|---|---|
| Abscess of Lungs, | | 1 | 1 | | | | |
| Apoplexy, | | 1 | 1 | | | | |
| Chronic Inflammation of Brain, | 1 | | 1 | | | | |
| Convulsions, | 4 | 1 | 5 | 4 | | | 1 |
| Consumption, | 7 | 8 | 15 | 1 | | | |
| Congestion of Lungs, | | 1 | 1 | | | | |
| Child Bed, | | 2 | 2 | | | | |
| Croup, | 3 | | 3 | 2 | | | |
| Congestion of Brain, | 1 | | 1 | | | | |
| Cancer of Breast, | | 1 | 1 | | | | |
| Cholera Morbus, | | 1 | 1 | | | | |
| Diarrhœa, | 5 | 1 | 6 | 1 | | 1 | |
| Disease of Brain, | 1 | | 1 | | | | |
| Dropsy, | 1 | | 1 | | | | |
| " of Brain, | 2 | 1 | 3 | | | | 1 |
| Drowned, | 5 | 2 | 7 | | | | |
| Delirium Tremens, | 2 | | 2 | | | | |
| Dentition, | 1 | | 1 | 1 | | | |
| Debility, | | 1 | 1 | | 1 | | |
| Fever, Bilious, | 1 | | 1 | | | | |
| " Typhoid, | 1 | 1 | 2 | | | | |
| " Typhus, | 1 | | 1 | | | | |
| " Scarlet, | 2 | | 2 | 1 | | | |
| " Puerperal, | | 1 | 1 | | | | |
| "Febris Nervosa," | | 1 | 1 | | | | |
| Fracture of Spine, | 1 | | 1 | | | | |
| Hydrocephalus, | 2 | | 2 | 1 | | 1 | |
| Insanity, | | 1 | 1 | | | | |
| Marasmus, | 1 | | 1 | | | | |
| Measles, | 1 | 1 | 2 | 1 | | | |
| Neglect, | 1 | 1 | 2 | 1 | 1 | | |
| Old Age, | 2 | 3 | 5 | | | | |
| Pneumonia, | 2 | 2 | 4 | | | | |
| Pleurisy, | 1 | | 1 | | | | |
| Puerperal Peritonitis, | | 1 | 1 | | | | |
| Premature Birth, | 1 | 1 | 2 | 1 | 1 | | |
| Suicide, | 1 | | 1 | | | | |
| Scalded, | 1 | | 1 | 1 | | | |
| Still-born, | 2 | | 2 | 2 | | | |
| Small-pox, | 1 | | 1 | 1 | | | |
| Syphilis, | | 1 | 1 | | | | |
| Tubercular Ulceration of Larynx, | 1 | | 1 | | | | |
| Unknown, | 8 | 8 | 16 | 5 | 6 | | |
| Uterine Hæmorrhage, | | 1 | 1 | | | | |
| Whooping Cough, | 1 | | 1 | | | | |
| Totals, | 65 | 44 | 109 | 23 | 9 | 2 | 2 |

NATIVITIES.—American, 40. German, 29. English, 4. Irish, 16. Swiss, 1. French, 2.

*of June,* 1855. By J. Root, M. D., Health Physician.

AGE.

| 2—5 | | 5—10 | | 10—15 | | 15—20 | | 20—25 | | 25—30 | | 30—40 | | 40—50 | | 50—60 | | 60—70 | | 70—80 | | 80—90 | | 90—100 | | 100 and upward. | | Not given. | |
|---|---|---|---|---|---|---|---|---|---|---|---|---|---|---|---|---|---|---|---|---|---|---|---|---|---|---|---|---|---|
| Male. | Female. | Male. | Female. | Male. | Female. | Male. | Female. | Male. | Female. | Male. | Female. | Male. | Female. | Male. | Female. | Male. | Female. | Male. | Female. | Male. | Female. | Male. | Female. | Male. | Female. | Male. | Female. | Male. | Female. |
| .. | .. | .. | .. | .. | .. | .. | .. | .. | .. | .. | .. | .. | 1 | .. | .. | .. | .. | .. | .. | .. | .. | .. | .. | .. | .. | .. | .. | .. | .. |
| .. | .. | .. | .. | .. | .. | .. | .. | .. | .. | .. | .. | .. | .. | .. | .. | .. | 1 | .. | .. | .. | .. | .. | .. | .. | .. | .. | .. | .. | .. |
| .. | .. | .. | .. | .. | .. | .. | .. | .. | .. | .. | .. | .. | .. | .. | .. | 1 | .. | .. | .. | .. | .. | .. | .. | .. | .. | .. | .. | .. | .. |
| .. | .. | .. | .. | .. | .. | .. | .. | .. | .. | .. | .. | .. | .. | .. | .. | .. | .. | .. | .. | .. | .. | .. | .. | .. | .. | .. | .. | .. | .. |
| .. | 1 | .. | .. | .. | 1 | .. | .. | 1 | 1 | 1 | 2 | .. | 1 | 3 | .. | .. | 2 | .. | .. | .. | .. | .. | .. | .. | .. | .. | .. | 1 | .. |
| .. | .. | .. | .. | .. | .. | .. | .. | .. | .. | .. | .. | .. | .. | .. | .. | .. | .. | .. | .. | .. | 1 | .. | .. | .. | .. | .. | .. | .. | .. |
| .. | .. | .. | .. | .. | .. | .. | .. | .. | .. | .. | .. | .. | 2 | .. | .. | .. | .. | .. | .. | .. | .. | .. | .. | .. | .. | .. | .. | .. | .. |
| 1 | .. | .. | .. | .. | .. | .. | .. | .. | .. | .. | .. | .. | .. | .. | .. | .. | .. | .. | .. | .. | .. | .. | .. | .. | .. | .. | .. | .. | .. |
| .. | .. | .. | .. | .. | .. | .. | .. | .. | .. | .. | .. | .. | .. | .. | .. | .. | .. | .. | .. | .. | .. | .. | .. | .. | .. | .. | .. | 1 | .. |
| .. | .. | .. | .. | .. | .. | .. | .. | .. | .. | .. | .. | .. | .. | .. | .. | .. | .. | .. | 1 | .. | .. | .. | .. | .. | .. | .. | .. | .. | .. |
| 1 | .. | .. | .. | .. | .. | .. | .. | .. | .. | .. | .. | .. | .. | .. | .. | .. | .. | .. | .. | .. | .. | .. | .. | .. | .. | .. | .. | .. | .. |
| 1 | .. | .. | .. | .. | .. | .. | .. | .. | .. | 1 | .. | .. | .. | .. | .. | 1 | 1 | .. | .. | .. | .. | .. | .. | .. | .. | .. | .. | .. | .. |
| .. | .. | .. | .. | .. | .. | .. | .. | .. | .. | .. | .. | 1 | .. | .. | .. | .. | .. | .. | .. | .. | .. | .. | .. | .. | .. | .. | .. | .. | .. |
| .. | .. | .. | .. | .. | .. | .. | .. | .. | .. | .. | .. | 1 | .. | .. | .. | .. | .. | .. | .. | .. | .. | .. | .. | .. | .. | .. | .. | .. | .. |
| 2 | .. | .. | .. | .. | .. | .. | .. | .. | .. | .. | .. | .. | .. | .. | .. | .. | .. | .. | .. | .. | .. | .. | .. | .. | .. | .. | .. | .. | .. |
| 1 | .. | 1 | 1 | .. | 1 | 1 | .. | 1 | .. | 1 | .. | .. | .. | .. | .. | .. | .. | .. | .. | .. | .. | .. | .. | .. | .. | .. | .. | .. | .. |
| .. | .. | .. | .. | .. | .. | .. | .. | .. | .. | .. | .. | .. | 1 | .. | .. | .. | .. | 1 | .. | .. | .. | .. | .. | .. | .. | .. | .. | .. | .. |
| .. | .. | .. | .. | .. | .. | .. | .. | .. | .. | .. | .. | .. | .. | .. | .. | .. | .. | .. | .. | .. | .. | .. | .. | .. | .. | .. | .. | .. | .. |
| .. | .. | .. | .. | .. | .. | .. | .. | .. | .. | .. | .. | .. | .. | .. | .. | .. | .. | .. | .. | .. | .. | .. | .. | .. | .. | .. | .. | .. | .. |
| .. | .. | .. | .. | .. | .. | .. | .. | .. | .. | .. | .. | 1 | .. | .. | .. | .. | .. | .. | .. | .. | .. | .. | .. | .. | .. | .. | .. | .. | .. |
| .. | .. | .. | .. | .. | .. | .. | .. | .. | 1 | 1 | .. | .. | .. | .. | .. | .. | .. | .. | .. | .. | .. | .. | .. | .. | .. | .. | .. | .. | .. |
| .. | .. | .. | .. | .. | .. | .. | .. | .. | .. | .. | .. | .. | .. | .. | .. | .. | .. | 1 | .. | .. | .. | .. | .. | .. | .. | .. | .. | .. | .. |
| .. | .. | .. | .. | 1 | .. | .. | .. | .. | .. | .. | .. | .. | .. | .. | .. | .. | .. | .. | .. | .. | .. | .. | .. | .. | .. | .. | .. | .. | .. |
| .. | .. | .. | .. | .. | .. | .. | .. | .. | .. | .. | 1 | .. | .. | .. | .. | .. | .. | .. | .. | .. | .. | .. | .. | .. | .. | .. | .. | .. | .. |
| .. | .. | .. | .. | .. | .. | 1 | .. | .. | .. | .. | .. | .. | .. | .. | .. | .. | .. | .. | .. | .. | .. | .. | .. | .. | .. | .. | .. | .. | .. |
| .. | .. | .. | .. | .. | .. | .. | .. | .. | .. | .. | .. | 1 | .. | .. | .. | .. | .. | .. | .. | .. | .. | .. | .. | .. | .. | .. | .. | .. | .. |
| .. | .. | .. | .. | .. | .. | .. | .. | .. | .. | .. | .. | .. | .. | .. | .. | .. | .. | .. | .. | .. | .. | .. | .. | .. | .. | .. | .. | .. | .. |
| .. | .. | .. | .. | .. | .. | .. | .. | .. | 1 | .. | .. | .. | .. | .. | .. | .. | .. | .. | .. | .. | .. | .. | .. | .. | .. | .. | .. | .. | .. |
| .. | .. | .. | .. | .. | .. | .. | .. | .. | .. | .. | .. | .. | .. | 1 | .. | .. | .. | .. | .. | .. | .. | .. | .. | .. | .. | .. | .. | .. | .. |
| .. | 1 | .. | .. | .. | .. | .. | .. | .. | .. | .. | .. | .. | .. | .. | .. | .. | .. | .. | .. | .. | .. | .. | .. | .. | .. | .. | .. | .. | .. |
| .. | .. | .. | .. | .. | .. | .. | .. | .. | .. | .. | .. | .. | .. | .. | .. | .. | .. | .. | .. | .. | .. | .. | .. | .. | .. | .. | .. | .. | .. |
| .. | .. | .. | .. | .. | .. | .. | .. | .. | .. | .. | .. | .. | .. | .. | .. | .. | .. | .. | 1 | .. | 2 | 1 | .. | 1 | .. | .. | .. | .. | .. |
| 1 | 1 | .. | .. | .. | .. | .. | .. | 1 | .. | .. | 1 | .. | .. | .. | .. | .. | .. | .. | .. | .. | .. | .. | .. | .. | .. | .. | .. | .. | .. |
| .. | .. | .. | .. | .. | .. | .. | .. | .. | .. | 1 | .. | .. | .. | .. | .. | .. | .. | .. | .. | .. | .. | .. | .. | .. | .. | .. | .. | .. | .. |
| .. | .. | .. | .. | .. | .. | .. | .. | .. | .. | .. | .. | .. | .. | .. | .. | .. | .. | .. | .. | .. | .. | .. | .. | .. | .. | .. | .. | .. | 1 |
| .. | .. | .. | .. | .. | .. | .. | .. | .. | .. | .. | .. | .. | .. | .. | .. | .. | .. | .. | .. | .. | .. | .. | .. | .. | .. | .. | .. | .. | .. |
| .. | .. | .. | .. | .. | .. | .. | .. | .. | .. | .. | .. | .. | .. | .. | .. | 1 | .. | .. | .. | .. | .. | .. | .. | .. | .. | .. | .. | .. | .. |
| .. | .. | .. | .. | .. | .. | .. | .. | .. | .. | .. | .. | .. | .. | .. | .. | .. | .. | .. | .. | .. | .. | .. | .. | .. | .. | .. | .. | .. | .. |
| .. | .. | .. | .. | .. | .. | .. | .. | .. | .. | .. | .. | .. | .. | .. | .. | .. | .. | .. | .. | .. | .. | .. | .. | .. | .. | .. | .. | .. | .. |
| .. | .. | .. | .. | .. | .. | .. | .. | .. | .. | .. | .. | .. | .. | .. | .. | .. | .. | .. | .. | .. | .. | .. | .. | .. | .. | .. | .. | .. | .. |
| .. | .. | .. | .. | .. | .. | .. | 1 | .. | .. | .. | .. | .. | .. | .. | .. | .. | .. | .. | .. | .. | .. | .. | .. | .. | .. | .. | .. | .. | .. |
| .. | .. | .. | .. | .. | .. | .. | .. | .. | .. | 1 | .. | .. | .. | .. | .. | .. | .. | .. | .. | .. | .. | .. | .. | .. | .. | .. | .. | .. | .. |
| .. | 1 | 1 | .. | .. | .. | .. | .. | .. | .. | 1 | .. | .. | .. | .. | .. | 1 | 1 | .. | .. | .. | .. | .. | .. | .. | .. | .. | .. | .. | .. |
| .. | .. | .. | .. | .. | .. | .. | .. | .. | .. | .. | .. | .. | .. | .. | 1 | .. | .. | .. | .. | .. | .. | .. | .. | .. | .. | .. | .. | .. | .. |
| .. | .. | 1 | .. | .. | .. | .. | .. | .. | .. | .. | .. | .. | .. | .. | .. | .. | .. | .. | .. | .. | .. | .. | .. | .. | .. | .. | .. | .. | .. |
| .. | .. | .. | .. | .. | .. | .. | .. | .. | .. | .. | .. | .. | .. | .. | .. | .. | .. | .. | .. | .. | .. | .. | .. | .. | .. | .. | .. | .. | .. |
| .. | .. | .. | .. | .. | .. | .. | .. | .. | .. | .. | .. | .. | .. | .. | .. | .. | .. | .. | .. | .. | .. | .. | .. | .. | .. | .. | .. | .. | .. |
| .. | .. | .. | .. | .. | .. | .. | .. | .. | .. | .. | .. | .. | .. | .. | .. | .. | .. | .. | .. | .. | .. | .. | .. | .. | .. | .. | .. | .. | .. |
| .. | .. | .. | .. | .. | .. | .. | .. | .. | .. | .. | .. | .. | .. | .. | .. | .. | .. | .. | .. | .. | .. | .. | .. | .. | .. | .. | .. | .. | .. |
| .. | .. | .. | .. | .. | .. | .. | .. | .. | .. | .. | .. | .. | .. | .. | .. | .. | .. | .. | .. | .. | .. | .. | .. | .. | .. | .. | .. | .. | .. |
| .. | .. | .. | .. | .. | .. | .. | .. | .. | .. | .. | .. | .. | .. | .. | .. | .. | .. | .. | .. | .. | .. | .. | .. | .. | .. | .. | .. | .. | .. |
| 7 | 4 | 3 | 1 | 1 | 2 | 2 | 1 | 3 | 3 | 7 | 4 | 4 | 5 | 4 | 1 | 4 | 5 | 2 | 2 | 0 | 3 | 1 | 0 | 1 | 0 | 0 | 0 | 2 | 1 |

Canadian, 2. Hollander, 1. Colored, 1. Country not given, 13.—Total, 109.

